# Brevetoxin Forms Covalent DNA Adducts in Rat Lung Following Intratracheal Exposure

**DOI:** 10.1289/ehp.11068

**Published:** 2008-03-24

**Authors:** Faisal F.Y. Radwan, John S. Ramsdell

**Affiliations:** 1 Marine Biotoxins Program, Center for Coastal Environmental Health and Biomolecular Research, National Oceanic and Atmospheric Administration/National Ocean Service, Charleston, South Carolina, USA; 2 Sohag University, Faculty of Science, Sohag, Egypt

**Keywords:** brevetoxin, DNA adducts, epoxidation, harmful algal bloom, *Karenia brevis*, lung, metabolism

## Abstract

**Background:**

Human exposure to brevetoxins produced by the red tide organism, *Karenia brevis*, is an increasing public health concern. Using *in vitro* exposure of rat liver cells to brevetoxin B (PbTx-2), the primary toxin product of *K. brevis*, we previously showed that it formed C_27,28_-epoxy brevetoxin metabolites capable of covalently binding to nucleic acids, a common initiation step for carcinogenesis.

**Objective:**

This study was undertaken to evaluate nucleic acid adduction in lung following *in vitro* and *in vivo* brevetoxin exposures.

**Methods:**

To clarify reactions of brevetoxin epoxide with DNA, we analyzed reaction products of PbTx-6 (a C_27,28_ epoxide metabolite of brevetoxin B) with nucleosides. We also analyzed adducts from nucleic acid hydrolysates of isolated rat lung cells treated with PbTx-2 or PbTx-6 *in vitro* and lung tissue from rats after intratracheal exposure to PbTx-2 or PbTx-6 at 45 μg toxin/kg body weight.

**Results:**

Our results indicate that PbTx-2 forms DNA adducts with cytidine after treatment of isolated lung cells, and forms DNA adducts with adenosine and guanosine after intratracheal exposure.

**Conclusions:**

These results are consistent with metabolic activation of highly reactive brevetoxin intermediates that bind to nucleic acid. These findings provide a basis for monitoring exposure and assessing the hazard associated with depurination of brevetoxin–nucleotide adducts in lung tissue.

*Karenia brevis*, formerly *Gymnodinium breve*, a brevetoxin-producing dinoflagellate, is recognized as the major harmful algae forming the red tide blooms in the Gulf of Mexico (Davis 1948). Two classes of brevetoxins were originally isolated from *K. brevis* by high pressure liquid chromatography (Risk et al. 1979). Structural analysis of these fractions identified two backbone structures, containing either 11 (brevetoxin-B) or 10 (brevetoxin-A) fused cyclic ether rings (Lin et al. 1981; Shimizu et al. 1986). Each backbone structure has similar functional groups and is subject to similar metabolic modifications (Wang et al. 2004). Brevetoxins bind to voltage-gated sodium channels to alter gating transitions interfering with the sensitive movements that transition the channel’s closed, open, and inactivated states (Catterall and Risk 1981). The initial actions of brevetoxins lead to nerve activation; within a few seconds of nerve activation, transient repetitive neuronal discharges are followed by action potential depression and eventually by a complete blockade of neuronal excitability (Huang et al. 1984).

The direct consumption of brevetoxin-laden shellfish causes the food poisoning referred to as neurotoxic shellfish poisoning (NSP) in humans (McFarren et al. 1965). NSP has become a rare phenomenon, even with the frequent occurrence of red tide events, in large part due to effective monitoring and management of shellfish harvest. However, inhalation of airborne brevetoxins at beaches remains common, and the occupational and recreational hazards have been the subject of recent investigations (Backer et al. 2003, 2005). Experimental exposure of brevetoxin aerosols was first demonstrated by Woodcock (1948), and in the last decade several studies have characterized the toxicokinetics and adverse effects of brevetoxins after intratracheal exposure of rats or sheep (Abraham et al. 2005; Benson et al. 1999). During this time, various animal and tissue explant models have been used to discriminate the molecular actions of brevetoxins on voltage-gated sodium channels in lung smooth muscle, autonomic nerve, and central respiratory centers (Ramsdell 2008). Exposure to brevetoxin-containing aerosols has also been shown to cause severe allergic reactions in asthmatic patients (Fleming et al. 2007; Kirkpatrick et al. 2006), but to date the adverse effects of direct exposure to the respiratory tract have not been found to be severe in healthy subjects.

A second potentially adverse mode of brevetoxin action may be oxidative damage as result of brevetoxin metabolism. Previous research has shown that the brevetoxin B (PbTx-2) is widely metabolized after systemic administration to rats, after exposure to freshly isolated rat hepatocytes, and after treatment with cDNA-expressed rat cytochrome P-450 enzymes (Radwan and Ramsdell 2006; Radwan et al. 2005). Metabolic reactions were observed on the A-ring, H-ring, and R-group of the terminal K-ring. They included hydrolysis (A-ring), epoxidation (H-ring), oxidation (R-group aldehyde), reduction (R-group aldehyde and α, β-unsaturated carbon), and peptide conjugation (R-group α,β-unsaturated aldehyde) (Figure 1). The epoxide metabolites are of interest because epoxides are highly reactive intermediates that react with nucleophilic sites to form covalent adducts with cellular macromolecules such as DNA and proteins (Koskinen and Plna 2000).

The large variability of phase I or II enzymes among animal species and their target organs defines a key question concerning organ-specific toxicity. Whether the actual target has the capacity to activate (or efficiently deactivate) chemicals is a key factor in susceptibility to toxin adduction. Lung is a target organ for the toxicity of most inhaled toxins. Occupational, accidental, or prolonged pulmonary exposure to elevated concentrations of brevetoxins is now the primary route for toxicity in humans during red tide blooms. Yet the mechanism by which lung cells metabolize brevetoxin to reduce its potential toxicity is still unknown. Results of several studies indicate that a variety of xenobiotic-metabolizing cytochromes [such as cytochrome P450 (CYP) 1A and CYP3A families] in the human lung and in lung-derived cell lines contributes to the *in situ* activation of pulmonary toxins (Castell et al. 2005). Lung cells also express phase II enzymes such as epoxide hydrolase, UGT1A (glucuronyl transferase), and GST-P1 (glutathione *S*-transferase), which largely act as detoxifying enzymes.

Adduct formation is a key step in inducing genotoxicity as a result of exposure to chemicals and metabolites that damage nucleic acid. The present study aimed to investigate brevetoxin adduction with nucleic materials in the lungs of exposed rats and thus to provide insight into DNA adduction by brevetoxins following metabolic activation. Detection of brevetoxin–nucleic acid adduction has pharmacological and risk assessment importance and should provide a foundation for biological monitoring of human exposure to such genotoxic environmental agents.

## Materials and Methods

### Materials

Brevetoxins PbTx-2 (MH^+^, *m/z* 895) and PbTx-6 (MH^+^, *m/z* 911), derived from *K. brevis* cultures [purity reported by vendor to be ≥ 95% by high performance liquid chromatography (HPLC)], were purchased from EMD Chemicals, Inc. (San Diego, CA, USA). Nucleosides guanosine and cytosine were purchased from Calbiochem (EMD Biosciences, Inc., San Diego, CA, USA). Total nucleic acid was isolated using DNeasy Tissue Kit produced by Qiagen Inc. (Germantown, MD, USA). Dulbecco’s modified Eagle medium (DMEM) was purchased from Invitrogen (Carlsbad, CA, USA). Rat lung cells (CCL-192/RFL-6) were purchased from the American Type Culture Collection (Manassas, VA, USA). All other analytical or molecular biologic grade chemicals used in this study were purchased from either Sigma Chemical Co. (St. Louis, MO, USA) or Fisher Scientific (Suwanee, GA, USA).

### Semisynthesis of nucleoside adducts with PbTx-6

Reaction mixtures (200 μL total volume) of PbTx-6 (200 μM) were incubated with aliquots of 50 mM equivalents of individual nucleosides prepared in 100 mM phosphate buffer at pH 7.4. The incubation was carried out in a shaking bath overnight at 37°C. The reaction mixture was centrifuged at 900 × *g* for 5 min to remove any particulates. We isolated the brevetoxin nucleoside adducts in the supernatant using a 500 mg C-18 cartridge (Varian; Palo Alto, CA, USA) previously conditioned with 3 mL methanol followed by 3 mL distilled water. Reactions were loaded, washed with 3 mL 10% methanol, and adducts were eluted with 5 mL 85% methanol. We dried and reconstituted adducts in 50% methanol for further radioimmunoassay (RIA) and HPLC/mass spectrometry (MS) analyses.

### *In vitro* treatment of lung cells with PbTx-2 and PbTx-6

We prepared toxins in methanol and diluted them in the culture medium (DMEM) so that the final methanol concentration was 1.5% (vol/vol); we then preincubated the preparation at 37°C for 10 min. Aliquots of rat lung cell culture suspension were transferred into new incubation wells and preincubated at 37°C for 10 min. We initiated toxin exposure by adding PbTx-2– or PbTx-6–enriched medium to a well containing cells. The final incubation mixture contained 35 μM of toxin and a cell count of approximately 1.1 × 10^6^ cells/mL. Control reactions were preformed by incubating cells in brevetoxin-free medium. We determined cell viability (> 86%) before exposure using the trypan blue (0.4%) exclusion test. All incubations were performed at 37°C for 18 hr in a 5% CO_2_ humidified atmosphere. Cells were pelleted by centrifugation at 1,500 × *g* for 10 min and immediately frozen at −80°C for further nucleic acid extraction.

### Rat intratracheal exposures to PbTx-2 and PbTx-6

Brevetoxin treatments were carried out at Charles River Laboratories Inc., (Horsham, PA, USA) using 12-week-old Charles River male rats [Crl:CD(SD)IGS BR VAF/Plus; 225–250 g body weight]. Animals were separated into three groups of five rats per group and identified using Monel self-piercing ear tags. Brevetoxins dissolved in 10% methanol were further diluted in physiological saline containing 0.01% Emulphor EL-620 (GAF Corp., New York, NY, USA). Rats in the treatment groups were administered PbTx-2 (group 1) or PbTx-6 (group 2) at 45 μg/kg body weight through intratracheal instillation. We determined the dosage by a preliminary range-finding study of the maximally tolerable dose for PbTx-2 as defined by labored breathing not greater than 30 min after exposure. Briefly, rats were anesthetized using 5% isoflurane in O_2_ and intubated intratracheally as described previously (Medinsky et al. 1986). The doses, in a volume of 0.15 mL saline, were instantaneously delivered directly to the lung by a Luer-Lok syringe through a 16-gauge catheter. Rats were killed 24 hr after treatment, and the lungs were collected immediately and kept frozen at −80°C until use. Control rats received brevetoxin-free vehicle solution (group 3). Animal use was conducted according to Good Laboratory Practice Procedures, and the animals were treated humanely and with regard for alleviation of suffering. The experimental protocol was approved by the Charles River Laboratories Institutional Animal Care and Use Committee.

### Nucleic acid extraction and digestion

We isolated nucleic acid from rat lung using Qiagen Genomic-tips, essentially as described by the manufacturer, with the following modifications. Cultured cells (~ 3.3 × 10^6^/test) were thawed and resuspended in 200 μL phosphate buffer (0.1 M). Lung tissues were minced into smaller pieces in a Petri dish containing Dulbecco’s phosphate-buffered saline (PBS) and 20 mM EDTA, pH 7.8, on ice. We transferred 25-mg pieces to a 1.5-mL centrifuge tube and rinsed them at least three times in PBS. Cells or lung tissues were initially digested with protease K for 4 hr at 65°C. The mixture was loaded into the DNeasy spin column placed in a 2-mL collection tube. Unbound substances were collected during washing process. We determined the purity of the eluted nucleic acid extract by the ratio of absorbance at 260 nm (A_260_) and A_280_ (the accepted purity ratios A_260_/A_280_ were ~ 1.85). We determined the concentration based on a solution containing 50 μg/mL of double-stranded DNA having an absorbency (optical density; OD) of 1.0 at a wavelength of 260. Extracts were stored at −80°C until use.

### Nucleic acid hydrolysis and adduct purification

Extracts were acid hydrolyzed in 0.1 N HCl at 75°C for 30 min (Hertzog et al. 1980). We then adjusted the pH to about 4.5 with 5 N NH_4_OH. The nucleic acid hydrolysates were redissolved in 10% methanol and loaded onto a previously conditioned SPE C-18 cartridge (500 mg; Varian). The cartridge was washed with 5 mL distilled water, and brevetoxin adducts were then eluted with 10 mL 85% methanol. The eluate containing brevetoxin nucleoside adducts was concentrated to dryness under nitrogen stream. We resuspended the dried adducts in 100 μL of 50% methanol for immediate RIA or liquid chromatography (LC)/MS analyses.

### RIA

We performed an RIA selective for toxins with the B-type backbone brevetoxin as described previously (Woofter et al. 2003). Tests were carried out in borosilicate glass tubes using antibodies raised in sheep immunized with PbTx-2–fetuin conjugate (Garthwaite et al. 2001). Assays were conducted in RIA buffer, a PBS containing 0.01% Emulphor-EL 620 with a final volume of 500 μL/test. Briefly, 5 μL of adduct extract, or PbTx-3 (the PbTx-2 terminal alcohol reduction product) standard set (from 0.01 to 1,000 nM) was incubated with 20 μL anti-PbTx antiserum (1:4,000) in RIA buffer for 1 hr at 25°C. Thereafter, 0.4 nM of the ^3^H-PbTx-3 tracer was added, and the incubation proceeded for another hour. Finally, we added 100 μL Sac-Cel (IDS Diagnostics, Fountain Hills, AZ, USA) to each of the assay tubes allowing the separation of the bound and unbound brevetoxin. The Sac-Cel mixture was filtered onto 25-mm diameter GF/B glass fiber filters (Whatman, Newton, MA, USA). The filters were immersed in 5 mL Scintiverse (Fisher, Suwannee, GA, USA) for 18 hr, and the radioactivity was counted on a Tri-Carb 3100TR liquid scintillation counter (Packard Bioscience Company, Meriden, CT, USA). Standard curves were constructed for each individual experiment, and results were expressed as PbTx-3 equivalents per microgram DNA in the original extract. Extracts from controls did not have measurable matrix interference. The assay limit of detection for RIA of PbTx-3 was about 0.5 ng/mL.

### LC/MS analyses

Adduct formation was analyzed using a Shimadzu VP HPLC system equipped with a Hypersil Beta-Basic C-18 column (1 × 50 mm, 3 μm; Thermo Electron Co., San Jose, CA, USA). The mobile phase consisted of water (A) and methanol (B) in a binary system, with 0.18% formic acid as an additive. The elution gradient was 45–75% B for 30 min, and the flow rate was 0.l mL/min. We analyzed adduct formation using the Q-STAR quadropole/time-of-flight mass spectrometer (Applied Biosystems/MDS SCIEX, Foster City, CA, USA). The mass spectrometer detected positive ions over the mass range *m/z* 300–1,400 or 100–1,400 atomic mass units (amu) at an orifice potential of 30 V. Selective molecular ions of nucleoside modified with brevetoxin, the molecular ions arising from the elimination of the ribose moiety from the parent compounds, and the loss of respective nucleobase were monitored. Ultraviolet chromatograms were also recorded at a wavelength of 260 nm.

## Results

### Semisynthesis of PbTx-6 nucleoside adducts

**Figure 2:**
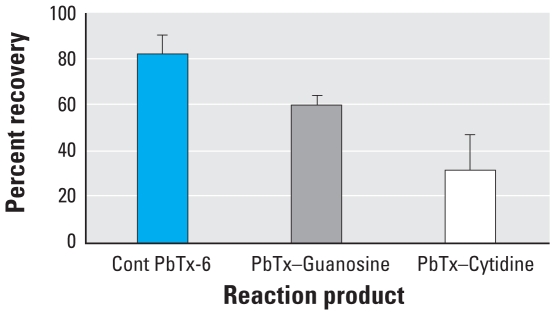
RIA of brevetoxin activity recovered from reaction products of PbTx-6 (mean ± SD) in the absence (Cont PbTx-6) and presence of guanosine (PbTx–Guanosine) or cytidine (PbTx–Cytodine). Incubation of PbTx-6 with individual nucleoside was carried out at 37°C for 18 hr, and brevetoxin products were separated using a C-18 cartridge.

**Figure 3:**
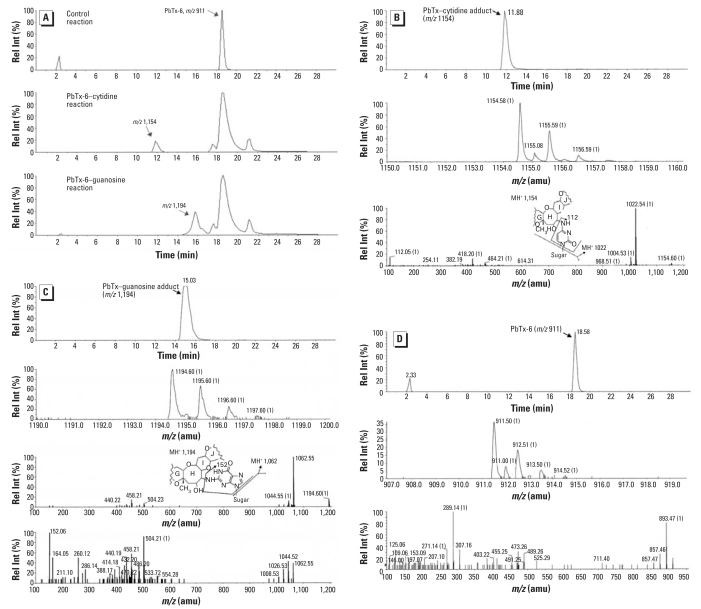
LC/MS analysis of semisynthesized brevetoxin nucleic acid adducts. Rel Int, relative intensity. (*A*) Extracted ion current (*m/z* 860–1,400) from total ion chromatograms for brevetoxin–cytidine, brevetoxin–guanosine, and unbound PbTx-6. (*B–D*) Extracted total ion chromatogram, mass spectra, and product for brevetoxin–cytidine (*B*), brevetoxin–guanosine (*C*), and unbound PbTx-6 (*D*). The brevetoxin-adduct fragment patterns shown in (*B*) and (*C*) depict a hypothetical nucleoside linkage to the epoxide to illustrate potential nucleoside fragmentation.

RIA of PbTx-6 extracted from the incubation mixture with nucleosides identified a reduction in recovered brevetoxin immuno-reactivity, consistent with the formation of brevetoxin nucleoside adducts (Figure 2). Figure 3A–C shows the LC/MS peak of nucleoside adducts that were not observed in control mixtures. Only a single peak of brevetoxin adduct was identified with each of these nucleosides. The molecular ion of each adduct indicated that one molecule of PbTx-6 was adducted to each nucleoside. Peaks had molecular ions corresponding to adduction products between PbTx-6 and nucleosides: retained at 11.8 min (*m/z* 1,154) for brevetoxin–cytidine (Figure 3A) and at about 15 min (*m/z* 1,194) for brevetoxin–guanosine adduct (Figure 3B). Unbound PbTx-6 (*m/z* 911) was retained at about 18.5 min, identical for both control and treatments (Figure 3C). Fragmentation of nucleoside adducts resulted in initial elimination of the ribose moiety from the parent compounds: *m/z* 1,154 to *m/z* 1,022 for brevetoxin–cytidine and *m/z* 1,194 to *m/z* 1,062 for brevetoxin–guanosine. The loss of respective nucleobase was monitored in both adducts giving rise to ions *m/z* 112 or *m/z* 152 for cytosine or guanine, respectively. Fragments of nucleobase imine were seen as *m/*z 124 (C=N^4^–) and *m/*z 164 (C=N^2^–) for cytosine or guanine, respectively. PbTx-6 was characterized by its daughter fragments *m/*z 857 and *m/z* 473 (Figure 3C).

### Adduct formation after *in vitro* exposure

**Figure 4:**
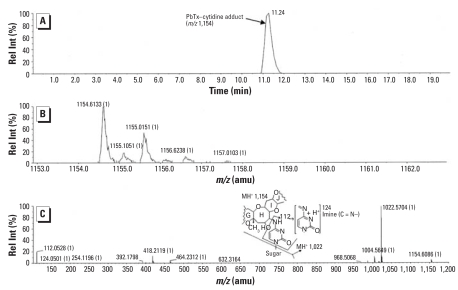
LC/MS of brevetoxin–nucleic acid adducts formed by reaction of PbTx-2 with isolated rat lung cells shown as extracted ion current (*m/z* 860–1,400) from (*A*) a total ion chromatogram, (*B*) mass spectra, and (*C*) product ion spectra. Rel Int, relative intensity. The spectra shows brevetoxin–cytidine adduct molecule (*m/z* 1,154) and products (*m/z* 1,022, *m/z* 124, and *m/z* 112) resulting from fragmentation of the nucleoside. The brevetoxin adduct fragment pattern shown depicts a hypothetical nucleoside linkage to the epoxide to illustrate potential nucleoside fragmentation.

Lung cells exposed to PbTx-2 showed an approximate 4-fold increase in the amount of adduct compared to PbTx-6 treatment. RIA showed 115.5 ± 31 pg PbTx-6 equivalents per microgram DNA for cells exposed to PbTx-2 and 34.1 ± 3 pg/μg DNA of cells exposed to PbTx-6. LC/MS analysis of extracts of PbTx-2 exposure revealed molecular ions corresponding to products between brevetoxin and cytidine. These ions included *m/z* 1,022 (brevetoxin-nucleobase after losing the sugar moiety) and cytosine *m/z* 112 and cytosine *N* ^4^ imine *m/z* 124 (Figure 4). Neither adduct parent molecule nor its legitimate sugar-free ion fragment was detected in PbTx-6 exposures.

### Adduct formation after *in vitro* exposure

**Figure 5:**
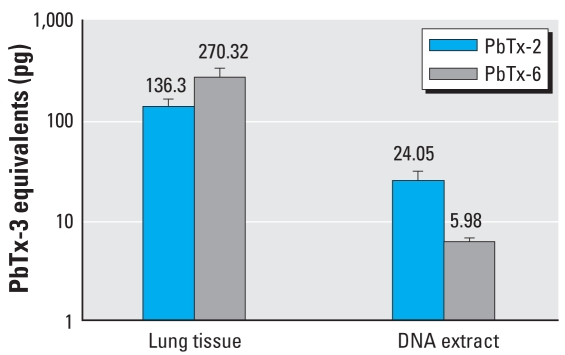
RIA of brevetoxin activity retained in lung tissues or associated with DNA extracts 24 hr after *in vivo* exposures to PbTx-2 or PbTx-6. Data shown are mean ± SD of PbTx-3 equivalents.

**Figure 6:**
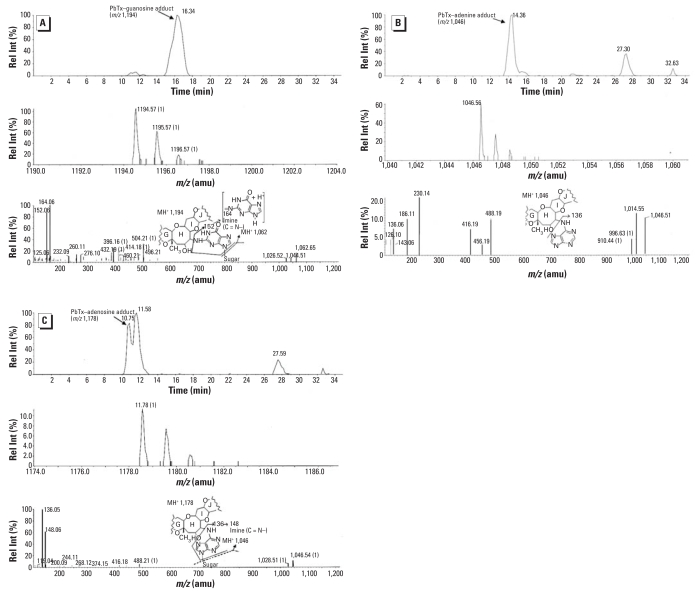
LC/MS of brevetoxin–nucleic acid adducts formed by brevetoxin reaction in rat lung after intratracheal exposure. Rel Int, relative intensity. (*A–C*) Extracted ion current (*m/z* 860–1,400) from total ion chromatograms, mass spectra, and product ion spectra for (*A*) brevetoxin–guanosine *m/z* 1,194, (*B*) brevetoxin–adenine *m/z* 1046, and (*C*) brevetoxin–adenosine *m/z* 1,178, respectively. The brevetoxin adduct fragment patterns shown in the spectra of (*A–C*) depict a hypothetical nucleoside linkage to the epoxide to illustrate potential nucleoside or nucleotide fragmentation.

A considerable amount of brevetoxin activity was retained in lung tissue 24 hr after exposure. An average of 136.3 ng PbTx-3 equivalents/g of lung tissue was measured for PbTx-2, while almost 2-fold more was measured in PbTx-6–exposed rats. RIA analyses of the nucleic acid hydrolysate showed about 4-fold greater adduct formation caused by PbTx-2 exposure (Figure 5). Further LC/MS analysis of the nucleic acid hydrosylate of PbTx-2–exposed lung revealed three molecular ions corresponding to brevetoxin adducts with nucleosides. The brevetoxin–guanosine adduct retained around 16 min (*m/z* 1,194) (Figure 6A), brevetoxin–adenine (*m/z* 1,046) retained at about 14 min (Figure 6B), and the brevetoxin–adenosine adduct retained at about 10.7 min (*m/z* 1,178; Figure 6C). Fragmentation spectra of nucleosidic adducts showed initial elimination of the sugar moiety to give daughter compounds: *m/z* 1,194 to *m/z* 1,062 for brevetoxin–guanosine and *m/z* 1,178 to *m/z* 1,042 for brevetoxin–guanosine. The loss of respective nucleobase was monitored in both adducts giving rise to ions *m/z* 152 or *m/z* 136 for guanine or adenine, respectively. Fragments of nucleobase imine were seen as *m/*z 164 (C=N^2^–) and *m/*z 148 (C=N^6^–). We could not detect adduct formation in hydrolysate extracts from PbTx-6 exposures using similar LC/MS conditions.

## Discussion

Brevetoxin, the red tide toxin, is well characterized for its neurotoxic toxic effects mediated by the voltage-gated sodium channel that are manifest in NSP (Ramsdell 2008). Respiratory exposure to seawater aerosols containing brevetoxins leads to a different constellation of effects (Backer et al. 2005), some which are consistent with activation of voltage-gated sodium channels, whereas others may involve inflammatory reaction pathways (Bossart et al. 1998). Brevetoxins can also lead to potential genotoxic effects. *In vitro* studies with human lymphocytes indicated that brevetoxins cause single-stranded and and double-stranded DNA breaks (Sayer et al. 2005), and a follow-up study in Chinese hamster ovary cells reported that brevetoxin causes chromosomal aberrations (Sayer et al. 2006).

Systemic absorption of brevetoxins by mammals leads to rapid metabolism to produce more readily excreted polar products (Radwan et al. 2005). The initial, or phase I reactions of brevetoxin include oxidation, reduction, hydrolysis, and epoxidation (Radwan and Ramsdell 2006). Epoxidation of the H-ring of brevetoxin B leads to a series of reactive intermediates that are capable of covalently binding with DNA or causing oxidative DNA damage. Formation of reactive intermediates is an initial step in the carcinogenic mechanisms of other well-known natural toxins such as aflatoxin (Groopman et al. 2005). In the present study we examined the subsequent step in this progression—namely, the formation of brevetoxin–nucleoside adducts in lung after intratracheal exposure to brevetoxins. Adduct formation is a key step in inducing genotoxicity as a result of exposure to nucleic acid-damaging chemicals and metabolites.

### Mechanism for brevetoxin–DNA adduct formation

We focused on brevetoxin B analogs; however, similar processes can be expected for brevetoxin A analogs. The initial brevetoxin B produced by the algae has a reactive α,β-unsaturated aldehyde extending from the terminal K-ring and has been designated as PbTx-2. PbTx-2 is also subject to epoxidation, in the 27, 28 position of the H-ring designated PbTx-6, which as been found both in algal cells (Chou et al. 1985) and as a metabolic product of PbTx-2 in liver cells (Radwan and Ramsdell 2006). In this article, we first described the semisynthesis of brevetoxin–nucleic acid adducts produced from reactions of PbTx-6 with cytosine and guanosine. Reaction of PbTx-6 with nucleosides was noted by a decrease in toxin activity in the assay mixture compared to the control, which suggests that nucleosidic adducts may have a lower affinity for brevetoxin antibody. LC/MS data provided structural evidence for the formation of covalent brevetoxin–cytidine and brevetoxin–guanosine adducts. The MS/MS spectrum of each adduct was identified by a parent [M + PbTx-6+H]^+^ ion, and two major fragment ions: [M + PbTx-6 – 132+H], probably formed by elimination of sugar moiety, and [M + PbTx-6 – 132 – 18+H], likely due to further loss of water. Elimination of respective nucleobase was observed as *m/z* 112 and *m/z* 152 for brevetoxin–cytidine and brevetoxin–guanosine, respectively. Respective nucleobase imine fragments *m/z* 124 and *m/z* 164 were also observed, indicating that adduction sites of nucleobases toward brevetoxin occurs at least in part on the exocyclic NH_2_ groups of the base moieties. LC/MS analysis did not yield sufficient structural information to confirm the site of nucleoside linkage to the H-ring epoxide.

### Characterization of brevetoxin–nucleoside adducts

Brevetoxin–nucleoside adducts were examined after *in vitro* exposure of brevetoxins to lung fibroblasts and after *in vivo* exposure of rats to brevetoxins via intratracheal administration. Two brevetoxin congeners were chosen for exposure, PbTx-6, which has the H-ring epoxide, and PbTx-2, which has been found to be metabolized to form the H-ring epoxide in liver cells. Both brevetoxin congeners yielded brevetoxin immunoreactiv-ity to purified nucleoside fractions recovered from exposed lung cells and lung tissue; however, in each case PbTx-2 yielded four times the activity found with PbTx-6 treatment. Extracted ion chromatograms identified brevetoxin adducts only with PbTx-2 treatment. Brevetoxin–cytidine adducts were detected in PbTx-2–exposed lung cells, but no adducts were found in the nucleic acid extracts of lung cells exposed to PbTx-6 under the same LC/MS conditions. Adducts of brevetoxin–guanosine *(m/z* 1,194) and brevetoxin–adenosine (*m/z* 1,178) were identified in the lung of rats exposed to PbTx-2 but not PbTx-6. These results are the first to establish the *in vitro* and *in vivo* formation of nucleic acid adducts after brevetoxin exposure.

Although the reactivity of PbTx-2, and not its epoxide PbTx-6, might be taken to mean that the epoxide is not the likely reactive group for conjugation with the nucleoside, further interpretation may indicate otherwise. PbTx-6 also has the K-ring α,β-unsaturated aldehyde, indicating that it should be as reactive for nucleoside conjugation by Michael addition as PbTx-2, which it is not. This suggests that epoxidation may be an intermediate step for PbTx-2 adduction. Epoxides are very aggressive alkylating agents of nucleophilic sites of nucleosides, and epoxides formed metabolically may have a higher probability of binding to DNA. Hence, an epoxide formed intracellularly by metabolism of PbTx-2 would have greater access to DNA than the epoxide of PbTx-6 when administered into the animal. DNA adduction to a metabolically formed epoxide of brevetoxin is also consistent with the report that both the single and double reduction of α,β-unsaturated aldehyde derivates of PbTx-2 caused DNA breaks in human lymphocytes as effectively as PbTx-2 (Sayer et al. 2005). Adduction of guanosine or adenosine to the epoxide would lead to the brevetoxin–guanosine and brevetoxin–adenosine masses *m/z* 1,194 and 1,178, respectively, identified in lung nucleic acid hydrolytes. However the K-ring R group α,β-unsaturated aldehyde is also likely to react with the nucleoside. For example, Michael addition with the nucleoside and subsequent oxidation of the aldehyde to carboxylic acid would also lead to the same mass adduct products. Additional analysis will be necessary to provide full structural assignments of the brevetoxin–nucleoside adducts in lung tissues. The structural assignment of the toxin nucleoside linkage, however, is less critical from a functional perspective, as multiple reaction products are bound to result from brevetoxin reactive intermediates. It is likely that more sensitive LC/MS techniques will identify additional brevetoxin–nucleic acid and brevetoxin–protein adducts in lung. This is important because, even though major toxin nucleic acid adducts product are necessary for the development of markers of exposure, less prominent adduct products are often responsible for genotoxic effects (Groopman et al. 2005).

### Potential for genotoxic effects following red tide exposure

This study presents the first evidence that brevetoxins form nucleoside adducts in lung tissue. We identified brevetoxin–guanosine *(m/z* 1,194) and brevetoxin–adenosine (*m/z* 1,178) as major products of hydrolyzed lung DNA after intratracheal exposure of rats. The finding of brevetoxin conjugated to guanosine and adenosine after hydrolysis of lung DNA is consistent with brevetoxin metabolism resulting in a reactive intermediate capable of reaching the nucleus from its site of metabolism in the mitochondria to react with DNA purines. DNA purine adducts are known to be unstable and to undergo depurination. The release of the nucleotide base adduct is an indication of a promutagenic lesion. Structurally, the lesion in the DNA is the result of an apurinic site that remains in the DNA base sequence. The identification of brevetoxin–nucleotide base (adenine) adduct (*m/z* 1,046) in addition to brevetoxin–nucleoside (adenosine) adduct (*m/z* 1,178) in rat lung tissue is consistent with the formation of promutagenic lesions after brevetoxin exposure. Promutagenic lesions resulting from depurination of aflatoxin–nucleoside adducts are susceptible to faulty repair, causing G to T transversions, which have been shown to result in inactivation of the TP53 tumor-suppressor gene in aflatoxin-associated hepatocarcinoma (Kensler et al. 2003).

### Potential risk of exposure to brevetoxin aerosols

The risk of brevetoxin adduct formation and resultant depurination is not known. Individuals are continually exposed to environmental agents capable of direct adduct formation as well as adduct formation through environmental agents that induce oxidative damage. It is possible that exposure to brevetoxins in aerosols, like other inhaled environmental genotoxic carcinogens, including polyacromatic hydrocarbons (Schoket 1999) and tobacco smoke (Wiencke 2002), add to the baseline levels of DNA adducts. Baseline levels of adducts in humans are estimated to be in the range of 0.1–1.0 adduct per 10^8^ unmodified DNA bases, and this burden can rise by four orders of magnitude in animals exposed to carcinogenic levels of genotoxic agents (Singh and Farmer 2006). A question that remains is whether inhalation exposure to brevetoxin aerosols leads to significant lung DNA depurination. Key to this question is the rate of brevetoxin adduction to DNA bases in lung and the extent of exposure.

The identification of brevetoxin–purine adducts now provides a foundation for biological monitoring of human exposure to such genotoxic environmental agents. The dose levels used in these *in vitro* and *in vivo* experiments were not meant to be reflective of human inhalation exposure to brevetoxins, which has been estimated at 3 and 20 ng/hr for red tide events in Florida (2003) and Texas (2000), respectively (Cheng et al. 2005; Pierce et al. 2005). Rather, the dosage was high, by several orders of magnitude, to optimize the search for toxin–nucleoside adducts. Dose–response studies are an important next step, and previous studies have shown that DNA adducts are formed on a linear basis to dose (Buss et al. 1990). However, most biological monitoring studies use readily obtained samples, such as white blood cells for DNA adducts, serum for albumin adducts or urine for nucleotide adducts. The release of the nucleotide adduct from target tissue DNA as a result of depurination leads to the adduct elimination in urine. Identification of urinary nucleotide adducts using LC/MS has provided a foundation for large-scale monitoring studies of several genotoxic agents (Shuker and Farmer 1992; Singh and Farmer 2006) and could prove useful for brevetoxins as well.

## Figures and Tables

**Figure 1:**
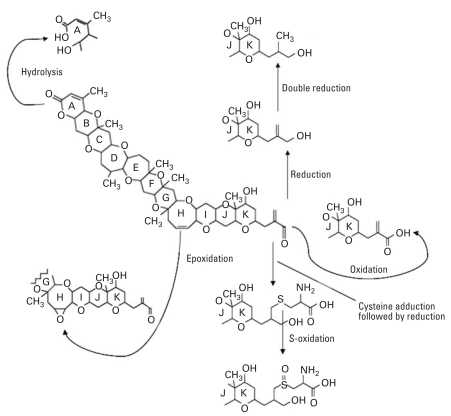
Schematic diagram showing the intermediary metabolites generated after *in vitro* metabolic bioactivation pathways of brevetoxin-B in rat liver cells.
